# Current Inflammatory Status of Monocyte Ratios in Bell’s Palsy

**DOI:** 10.7759/cureus.57770

**Published:** 2024-04-07

**Authors:** Serkan Serifler, Fatih Gul, Kadir sinasi Bulut, Gökhan Yalcıner, Ozgenur Kocak, Mehmet Ali Babademez

**Affiliations:** 1 Otolaryngology-Head and Neck Surgery, Duzce Ataturk State Hospital, Duzce, TUR; 2 Otolaryngology-Head and Neck Surgery, Ankara City Hospital, Ankara, TUR; 3 Otolaryngology-Head and Neck Surgery, Yildirim Beyazit University Faculty of Medicine, Ankara, TUR; 4 Otolaryngology-Head and Neck Surgery, Ankara Haymana State Hospital, Ankara, TUR; 5 Otolaryngology-Head and Neck Surgery, Ankara Bilkent City Hospital, Ankara, TUR; 6 Otolaryngology, Zonguldak Caycuma State Hospital, Zonguldak, TUR

**Keywords:** monocytes to lymphocytes ratio, inflammation, high‐density lipoprotein (hdl), monocyte, bell’s palsy

## Abstract

Objective: This study aims to investigate the contribution of monocyte/high-density lipoprotein (HDL) ratio (MHR) and monocyte/lymphocyte ratio (MLR) to the inflammatory process and the severity and prognosis of the disease in patients with Bell’s palsy.

Materials and methods: The study was designed retrospectively by analyzing our electronic database. A study group consisted of 48 patients who were referred to our clinic with Bell’s palsy between January 2018 and June 2020. The control group consisted of 45 healthy individuals. Monocyte, HDL, neutrophil, lymphocyte, and platelet values were recorded. The hematological parameters obtained from the blood tests of the patients in the study group at the time of admission were statistically compared with the values in the control group. Radiologic images were also collected.

Results: The MHR value of the study group was 12.85±1.02, while the MHR value of the control group was 12.29±1.33, and it showed a statistically significant difference (p=0.027). However, no statistically significant difference between the groups was found in other parameters, including MLR, neutrophil/lymphocyte ratio (NLR), and platelet/lymphocyte ratio (PLR). A positive correlation was found between the MHR value and the House-Brackmann stage. The NLR value of the patients who showed contrast enhancement in facial nerves on MRI was found to be statistically significant compared to those without contrast enhancement.

Conclusion: High MHR values in patients with Bell’s palsy support the role of inflammatory and ischemic processes in etiopathogenesis. Further studies are needed to confirm our results in a multi-center manner with larger patient populations.

## Introduction

About 90% of peripheral facial paralysis is idiopathic. Its pathogenesis is yet to be fully elucidated [1]. In many studies investigating the etiopathogenesis of Bell’s palsy, factors such as inflammation, viral infection, and microvascular insufficiency are seen to play a role. In inflammation theory, the nerve gets thickened and pinched in the fallopian canal due to edema. Secondly, viral infections may cause inflammation. In the last theory, microvascular insufficiency may cause a facial nerve injury due to ischemia or thrombus in the vasa nervosum of the nerve [2].

Although Bell’s palsy is a self-limiting disease that usually results in full recovery, there may be times when it is necessary to predict the severity and prognosis of the disease [3]. Since the topographic and electrophysiological tests used in these situations are laborious and costly, simpler and cheaper tests are needed. For this purpose, it has been suggested that neutrophil/lymphocyte ratio (NLR), platelet/lymphocyte ratio (PLR), and thiol/disulfide markers may be effective in the follow-up or prognosis of facial paralysis [4,5]. However, none of these parameters have been established in the current practice of otologists in the follow-up and prognosis of Bell’s palsy.

In recent years, the role of high-density lipoprotein (HDL), monocyte, lymphocyte, monocyte/HDL ratio (MHR), and monocyte/lymphocyte ratio (MLR) in inflammation has been investigated as oxidative stress markers [6]. Based on the inhibitory effect of HDL on monocytes, the relationship between atherosclerosis and the value of MHR, which is commonly used in cardiovascular diseases, has been investigated [6,7]. MLR is also used as a marker to show inflammation in rheumatologic and cancer patients [8]. In otolaryngological diseases, the effect of MHR has only been investigated in sudden sensorineural hearing loss and obstructive sleep apnea [9]. All these recent studies showed that MHR and MLR were indicative of systemic inflammation, similar to other parameters.

In the literature, several studies investigated hematologic parameters, especially NLR, in Bell’s palsy [10]. In addition to the parameters used, we aimed to investigate the relationship between MHR, MLR, and the disease. According to our literature knowledge, this is the first study investigating the effect of MHR and MLR on the severity of the disease and the prediction of the prognosis of Bell’s palsy.

## Materials and methods

This clinical study was conducted retrospectively on patients diagnosed with Bell’s palsy between January 2018 and June 2020. The study protocol was approved by the local ethics committee (Decision No.: 26379996/44) and was performed in accordance with the ethical rules and principles of the Declaration of Helsinki.

Patients under the age of 18 and over 65 years, those with hematological diseases, a history of cancer, acute coronary artery disease, chronic kidney disease, rheumatological disease, inflammatory soft tissue disease, and those using corticosteroid therapy were excluded from the study. So, 48 patients diagnosed with Bell’s palsy were included in the study. The control group included 45 healthy individuals with no evidence of otologic disease or no history of facial paralysis.

The data of all patients was scanned. Their examination findings and comorbidities, such as diabetes mellitus and hypertension, were recorded. A complete blood count and biochemical tests, including blood lipids and coagulation tests, were also recorded. Radiological images of the study group were obtained, and the presence of pathological enhancement in the facial nerve was noted on an MRI. In addition, the facial paralysis stage of the patients in the study group was recorded according to the House-Brackmann (HB) scale at the time of admission. All of the patients were administered with the same therapeutic protocol, which included prednisone 1 mg/kg/day, tapered, and stopped in two weeks. All patients in the study group had been followed for six months.

MHR was calculated as a simple ratio between the absolute monocyte and the absolute HDL value. MLR was also calculated as a simple ratio between the absolute monocyte and the absolute lymphocyte counts. The total monocyte count, leukocyte count, lymphocyte count, platelet count, HDL values, NLR, and PLR of the study and control groups were recorded.

Statistical analysis

IBM Corp. Released 2019. IBM SPSS Statistics for Windows, Version 26.0. Armonk, NY: IBM Corp. was used for the statistical analysis. Numerical data were presented as the mean ± standard deviation. The Shapiro-Wilk test was used to examine the normal distribution of the data. A student's t-test was used for comparisons between the patient and control groups and also between the contrast-enhancing and non-enhancing groups. A Pearson correlation analysis test was used to examine the correlation between numerical data. The interpretation of the Pearson correlation analysis is as follows: r=0.00-0.29 "weak," 0.30-0.49 "low," 0.50-0.69 "medium," 0.70-0.89 "strong," and 0.90-1 "very strong" [11]. The receiver operating characteristic (ROC) analysis was performed to determine the cutoff values, sensitivity, and specificities of NLR in predicting Bell's palsy and MHR in predicting contrast enhancement. P<0.05 was considered statistically significant.

## Results

The study group consisted of 48 patients (29 males and 19 females) with a mean age of 47 years, while the control group was 45 patients (26 males and 19 females) with a mean age of 43.8 years. Age and gender did not show a statistically significant difference between the groups (p>0.05). The demographic data of the study and control groups are shown in Table 1.

**Table 1:** Demographic clinical data of the study and control groups HDL: high-density lipoprotein; HB: House-Brackmann

	Study group (n=48)	Control group (n=45)	p-value
Age, y	47.44±12.89	43.89±13.56	0.199
Gender, n, (%)			0.798
Female	19 (39.6%)	19 (42.2%)	
Male	29 (60.4%)	26 (57.8%)	
Facial paralysis, n, (%)			
right	22 (45.8%)		
left	26 (54.2%)		
Status of cigarette (+), n, (%)	16 (33.3%)	12 (27.6%)	0.483
Hypertension (+), n, (%)	12 (25.0%)	9 (20%)	0.562
Diabetes mellitus (+), n, (%)	10 (20.8%)	6 (13.3%)	0.334
House-Brackmann stage, n			
Grade 2	4		
Grade 3	22		
Grade 4	13		
Grade 5	7		
Grade 6	2		
Monocyte count (×10^9^/μl)	458.88±92.98	437.24±107.35	0.301
Neutrophil count (×10^9^/μl)	6633.93±1456.08	6416.68±1477.25	0.477
Lymphocyte count (×10^9^/μl)	1996.06±398.60	1957.97±426.53	0.657
Platelet count(×10^9^/μl)	265.73±113.65	261.53±120.59	0.863
HDL (mg/dl)	35.89±7.76	35.96±9.72	0.967

Although the mean monocyte, neutrophil, platelet, and lymphocyte values of the study group were higher than those of the control group, there were no statistically significant differences (p=0.301, p=0.477, p=0.657, and p=0.863, respectively). Although the HDL value was higher in the control group when compared to the study group, it did not show a statistically significant difference (p=0.967). A statistically significant difference was observed between the study group and the control group in terms of MHR value (p=0.027), but no statistically significant difference was observed between the two groups in terms of MLR, NLR, and PLR values (Table 2).

**Table 2:** Comparison of hematological data of study and control groups HDL: high-density lipoprotein; MHR: monocyte/HDL ratio; MLR: monocyte/lymphocyte ratio; NLR: neutrophil/lymphocyte ratio; PLR: platelet/lymphocyte ratio

	Study group (n=48)	Control group (n=45)	p-value
MHR	12.85 ± 1.02	12.29 ± 1.33	0.027
MLR	0.23 ± 0.01	0.22 ± 0.02	0.107
NLR	3.31 ± 0.27	3.27 ± 0.25	0.434
PLR	131.34 ± 43.63	135.09 ± 60.62	0.732

The NLR values in the study group showed a statistically significant difference in the group showing enhancement in MRI compared to those without enhancement (p=0.004). However, there were no statistically significant differences between MRI and MHR, MLR, and PLR (Table 3).

**Table 3:** Contrast enhancement of facial nerves in MR images of the patients in the study group HDL: high-density lipoprotein; MHR: monocyte/HDL ratio; MLR: monocyte/lymphocyte ratio; NLR: neutrophil/lymphocyte ratio; PLR: platelet/lymphocyte ratio.

n= 48	Contrast Enhancement (+) Mean±SD	Contrast Enhancement (-) Mean±SD	p-value
MHR	13.01 ± 0.9	12.72 ± 1.1	0.337
MLR	0.22 ± 0.01	0.23 ± 0.01	0.515
NLR	3.44 ± 0.23	3.21 ± 0.26	0.004
PLR	129.4 ± 36.89	132.85 ± 48.88	0.789

When the relationship between the HB stage and blood parameters was examined, a positive correlation was found between the MHR and the HB stage (p<0.01). There were no correlations between the HB stage and NLR, MLR, or PLR (Table 4).

**Table 4:** Correlation between House Brackmann Stage and MHR MHR: monocyte/HDL ratio, NLR: neutrophil/lymphocyte ratio

n=48	Mean±SD	Pearson's correlation coefficient	p-value
MHR	12.85 ± 1.02	0.589	<0.01
House-Brackmann Stage	3.6 ± 1.02	-	-

**Figure 1:**
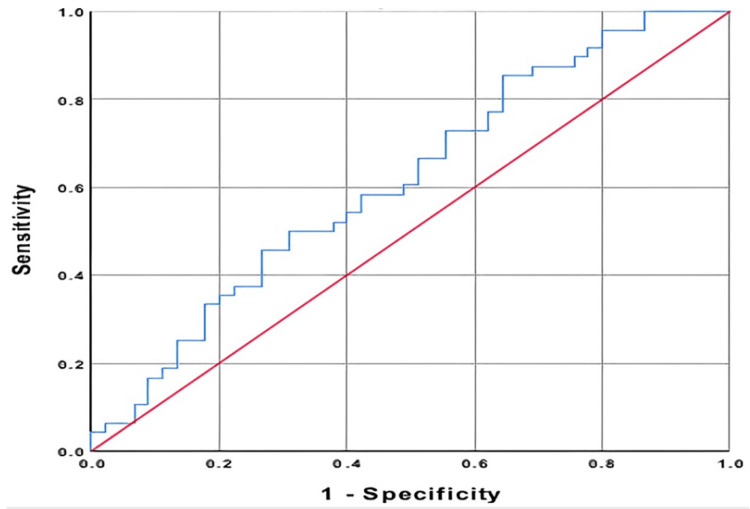
Monocyte/HDL ratio HDL: high-density lipoprotein.

**Figure 2:**
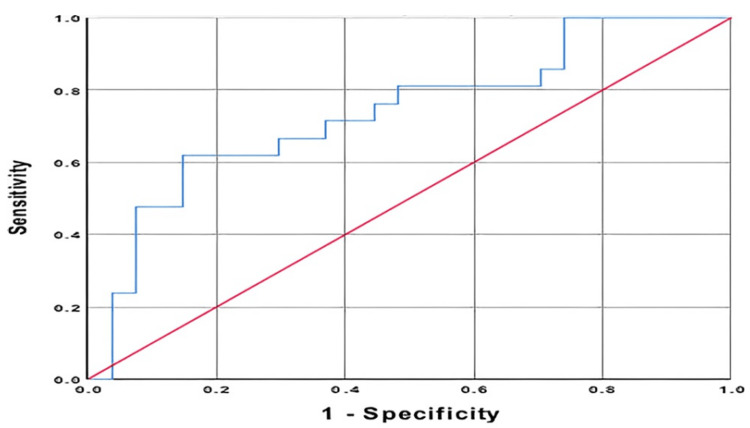
Neutrophil/lymphocyte ratio

ROC analysis was performed to measure the contribution of the MHR value in the diagnosis of Bell's palsy and the contribution of the NLR value in predicting contrast enhancement on MRI. A sensitivity of 58.3% and a specificity of 57.8% were found, with a cutoff value of 12.6 for MHR. When the cutoff value of NLR was taken to be 3.27, a sensitivity of 71.4% and a specificity of 63.0% were determined in predicting the enhancement of the facial nerve in MR images (Tables 5, 6; Figures 1, 2).

**Table 5:** Sensitivity and specificity of MHR in the diagnosis of Bell’s Palsy MHR: monocyte/HDL ratio

n=93	Cut-off	Area under the curve (AUC)	Standard error	95% confidence interval		Sensitivity	Specificity
				Lower bound	Upper bound		
MHR	12.6	0.616	0.058	0.501	0.730	58.3%	57.8%

**Table 6:** Sensitivity and specificity of NLR for contrast enhancement of facial nerves in MRI NLR: neutrophil/lymphocyte ratio

n=48	Cut-off	Area under the curve (AUC)	Standard error	95% confidence interval		Sensitivity	Specificity
				Lower bound	Upper bound		
NLR	3.27	0.737	0.074	0.592	0.882	71.4%	63.0%

## Discussion

Recently, MHR is believed to be a new cardiovascular prognostic marker indicating inflammation and oxidative stress. Some studies have shown that MHR is associated with inflammation, atherosclerosis, microvascular dysfunction, and coronary blood flow [6,7,12]. In the histopathological examination of the herpes simplex virus (HSV), which has been blamed for the etiology of Bell’s palsy, it has also been found in atherosclerotic plaques [13,14]. Physiopathologies contributing to Bell’s palsy may also be associated with the development of cardiovascular events such as acute myocardial infarction, as both may share common pathogenic pathways associated with HSV infection [15]. Underlying microcirculation insufficiencies in Bell’s palsy may occur due to atherosclerotic plaques. The monocyte count has already been shown to be an important and independent factor in the formation and development of atherosclerotic plaques. On the other hand, HDL has a vasoprotective role by preventing LDL oxidation, which has an important role in the formation of atherosclerotic plaque due to its anti-inflammatory, antithrombotic, and antioxidant effects [16]. Since similar factors may play a role in the etiopathogenesis of Bell’s palsy, we believe that MHR may be associated with the stage and prognosis of the disease. The most important feature of our study is that it is the first study to examine this issue for Bell’s palsy.

The statistically significant results of MHR suggest that it can be used as a marker indicating the inflammatory process as well as atherosclerotic plaques in the underlying pathophysiology of the disease. We investigated the relationship between MHR and the stage of the disease at the time of presentation to investigate the hypothesis that the same atherosclerosis may be caused by microvascular circulatory disorders in the facial nerve’s vaso nervosum. Since Pearson's correlation coefficient was found to be 0.589, it was observed that there was a moderately statistically significant relationship between MHR and the severity of paralysis. It is thought that MHR can be used as an auxiliary marker for staging in cases where there is doubt about clinical staging. As a result of these data, it is possible to interpret that the stage of the disease will increase as the severity of the inflammation caused by microvascular circulation insufficiency and atherosclerosis increases. The success of MHR in diagnosing facial paralysis was evaluated to be poor, with the AUC found to be 0.616, the sensitivity as 58.3%, and the specificity as 57.8%. Although MHR showed a statistically significant increase in the diagnosis of patients, it was not seen as a sufficient standalone laboratory test to diagnose facial paralysis.

MLR had previously been shown to be used as a systemic inflammation marker in rheumatological diseases and cancer [8,17]. Monocytes are stimulated with the onset of inflammation, and lymphocyte count decreases with inflammation, atherosclerosis, and plaque formation. Consequently, an increase in the MLR may be expected during systemic inflammation and cardiovascular events [18]. Based on this, we evaluated the MLR in Bell's palsy. However, unlike MHR, no statistically significant difference was observed for MLR in patients with facial paralysis compared to the control group.

We also examined NLR and PLR values, which were accepted inflammation parameters, but we could not find a significant difference between the study group and the control group. This situation did not coincide with other studies in the literature. Bucak et al. found higher NLR values in patients with facial paralysis [19]. Özler et al. investigated the relationship between NLR and disease stage and found a positive correlation between NLR and disease stage [10]. In our study, no significant correlation was found between the NLR and the stage of presentation. Kiliçkaya et al. investigated whether the NLR value could be used to evaluate the prognosis of the disease in facial paralysis, but they could not comment on the prognosis since most of the patients in the study showed complete recovery [20]. However, Wasano et al. were able to make a poor prognostic factor interpretation for NLR since there was a patient group that did not improve in their study, and the NLR value was statistically high in this group [21]. In our study, we could not interpret the prognosis for any parameter because all patients improved during the follow-up period.

Individuals with normal facial nerve functions might also have weak contrast enhancement, but the patients with Bell's palsy had seen a stronger pathological enhancement in MRI [22]. So, we evaluated the relationship between pathological enhancement and inflammatory parameters in MRI, and we found that only NLR was higher in the group with enhancement. This relationship was previously reported by Kum et al. [23]. We used the ROC curve to evaluate its diagnostic success. The success of NLR in determining facial nerve edema was evaluated as a medium, with the AUC found to be 0.737, sensitivity as 71.4%, and specificity as 63%. NLR can be used as an auxiliary laboratory test to MRI to determine the degree of edema in the facial nerve. In addition, we evaluated a relationship between pathological enhancement status and stage in MRI, and we could not find a significant result. If we could detect a relationship here, we could assume that the more sophisticated disease caused a more severe disease, and this was reflected in the radiological images.

Our study has a few limitations. To evaluate, in terms of prognosis, blood parameters should be checked periodically during the disease. So, this requires a prospective approach. Since our study is preliminary, our results would be a guide for future prospective studies. Second, a precise interpretation based on the prognosis could not be made as all patients improved during the follow-up period in our study. Therefore, individuals with partial or no improvement are required for the assessment of the prognosis.

## Conclusions

The present study is the first to investigate the association between MHR and MLR values and Bell’s palsy. The results showed statistically significant differences in the MHR between the study and control groups, as well as a positive correlation between the MHR and the HB stage. Additionally, the presence of contrast enhancement in the facial nerve on MRI was associated with statistically significant differences in the NLR. The conclusions drawn from these results suggest that high MHR values support the role of inflammation and ischemic processes in the development of Bell's palsy and that NLR may be used as an auxiliary test to determine the degree of edema in the facial nerve. However, it is noted that further studies with larger patient populations and a prospective design are needed to confirm these findings and evaluate the relationship between hematologic parameters and prognosis.
